# Facile Synthesis of Heterogeneous Indium Nanoparticles for Formate Production via CO_2_ Electroreduction

**DOI:** 10.3390/nano13081304

**Published:** 2023-04-07

**Authors:** Ana Cristina Pérez-Sequera, Manuel Antonio Diaz-Perez, Mayra Anabel Lara Angulo, Juan P. Holgado, Juan Carlos Serrano-Ruiz

**Affiliations:** 1 Materials and Sustainability Group, Department of Engineering, Universidad Loyola Andalucía, Avda. de las Universidades s/n, 41704 Dos Hermanas, Spain; 2 Instituto de Ciencia de Materiales de Sevilla and Departamento de Química Inorgánica, CSIC-Univ de Sevilla, Av. Américo Vespucio, 49, 41092 Seville, Spain

**Keywords:** indium catalysts, CO_2_ electroreduction, carbon-supported materials, heterogeneous catalysts, formate production, CO_2_ conversion

## Abstract

In this study, a simple and scalable method to obtain heterogeneous indium nanoparticles and carbon-supported indium nanoparticles under mild conditions is described. Physicochemical characterization by X-ray diffraction (XRD), X-ray photoelectron microscopy (XPS), scanning electron microscopy (SEM) and transmission electron microscopy (TEM) revealed heterogeneous morphologies for the In nanoparticles in all cases. Apart from In^0^, XPS revealed the presence of oxidized In species on the carbon-supported samples, whereas these species were not observed for the unsupported samples. The best-in-class catalyst (In_50_/C_50_) exhibited a high formate Faradaic efficiency (FE) near the unit (above 97%) at −1.6 V vs. Ag/AgCl, achieving a stable current density around −10 mA·cm_geo_^−2^, in a common H-cell. While In^0^ sites are the main active sites for the reaction, the presence of oxidized In species could play a role in the improved performance of the supported samples.

## 1. Introduction

The ever-increasing human population growth rate has increased the world energy demand, leading to higher CO_2_ emissions since a high percentage of the energy demand is still supported by fossil fuels [1]. The unceasing rise of the concentration of carbon dioxide, a powerful greenhouse gas, into the atmosphere is affecting the natural systems balance, leading to an acceleration of global warming [2]. In order to mitigate the impact of carbon dioxide emissions, several technologies have been developed [3,4,5,6,7]. Among them, electrochemical techniques able to upgrade carbon dioxide to value added chemicals have demonstrated to be a promising solution owing to their mild operating conditions and good performance [8]. By means of the electrochemical carbon dioxide reduction reaction (CO_2_RR), CO_2_ dissolved in aqueous medium can be converted into fuels and value-added chemicals [9], including carbon monoxide (CO), methanol (CH_3_OH), methane (CH_4_) and formic acid/formate (CHOOH), and even C_2+_ products such as ethanol (CH_3_CH_2_OH), acetic acid (CH_3_COOH), ethylene (C_2_H_4_) and n-propanol (CH_3_CH_2_CH_2_OH) [10,11,12,13]. Formic acid, an important chemical thoroughly employed in the industry, has gained a great attention recently due its potential use as hydrogen storage compound in fuel cells applications [14]. However, the production of formic acid is limited by a sluggish kinetic due to the high energy required to activate the CO_2_ molecule to the intermediate CO_2_^•−^. Moreover, the hydrogen evolution reaction (HER) represents another limitation due to its competitive equilibrium onset potential (close to that of CO_2_RR), leading to low products selectivities [15,16]. To overcome these barriers, highly active, stable and selective catalysts capable of suppressing HER are necessary [17,18,19]. In this sense, low toxicity and environmentally friendly transition metals such as indium (In) have demonstrated good electrocatalytic behavior as cathode material in the CO_2_RR since they combine high HER overpotentials and good selectivity to formate [20]. Nevertheless, indium-based materials typically present low current densities at low overpotentials and poor stability, exhibiting electrocatalytic activity decays after 2 h of electrolysis [21]. Hence, to improve the electrocatalytic performance and considering the rich structure sensitivity of the CO_2_RR, engineered indium-based catalysts have been synthesized with diverse shapes and morphologies including nanoparticles [22], nanocrystals [23], nanobelts [24], nanosheets [25], nanowires [26], nanocubes [27] and dendrites [28,29,30]. Thus, the morphology and shape of the indium particles can determine the number of edges, corners and plain sites, which can lead to enhancements on the activity, selectivity, stability and electronic properties [21,27]. Furthermore, the performance of the catalytic materials can be also improved by generating heterostructures or the introduction of structure defects (e.g., vacancies). These defects can modify the electronic properties of the catalyst surface, resulting in lower energy barriers [31,32,33,34]. For instance, Yang et al. achieved enhanced CO_2_RR activity by generating structure defects on synthesized In/In oxide heterostructures, resulting in formate selectivities as high as 93% at −0.9 V vs. reversible hydrogen electrode (RHE) in a 0.5 M KHCO_3_ solution [35]. Abundant carbon-based materials with tunable morphology, large surface area, porosity and high mechanical stability are optimal metal supports for the CO_2_RR [21,36]. In this regard, Rabiee et al. achieved high faradaic efficiencies (FE) to formate (70–77%) at −1.6 V vs. SCE in 0.5 K_2_SO_4_ over In(OH)_3_ nanocubic mesoporous particles supported on carbon black [27]. Despite the latest advances made in the development of active In catalysts for the CO_2_RR, the synthesis routes employed often consist on several complex steps under nonstandard conditions, thereby hindering scale up to industrial applications. Thus, it is highly desirable to develop catalyst synthesis methodologies with low complexity to ensure the economic viability of the overall process.

In this work, we describe a facile synthesis procedure to obtain indium nanoparticles (In NPs) and carbon-supported In NPs, and their performance was evaluated as CO_2_RR cathodes. In NPs were synthesized by a simple two-step method and subsequently washed with different mild reagents with the aim to optimize the behavior of the material. Moreover, In NPs were supported on carbon black at different mass ratios (In_20_/C_80_ and In_50_/C_50_). The as-obtained NPs were fully characterized by physicochemical techniques such as X-ray diffraction (XRD), X-ray photoelectron spectroscopy (XPS), scanning electron microscopy (SEM), transmission electron microscopy (TEM) and high-resolution TEM (HR-TEM). In addition, electrochemical techniques such as cyclic voltammetry and bulk electrolysis were applied to study the electroactivity of the catalysts in the CO_2_RR. The optimum catalyst obtained by the proposed method (In_50_/C_50_) achieved a stable current density above −10.6 mA·cm_geo_^−2^ at −1.6 V vs. Ag/AgCl with a formate FE as high as 97%. The presence of surface oxidized species and the support-catalyst interactions could be related with the superior behavior of this catalyst. Our results provide an easy and affordable synthetic methodology to produce highly efficient and stable indium-based nanoparticles with excellent formate selectivity.

## 2. Materials and Methods

### 2.1. Reagents and Materials

Indium (III) chloride (InCl_3_, 99.999%), polyvinylpyrrolidone (PVP, K30, Mw~55.000), sodium borohydride (NaBH_4_, 99%), Nafion™ perfluorinated resin solution dispersion (5 wt. % in mixture of lower aliphatic alcohols and water) and indium foil (CAS N° 744-74-6, 0.1 mm thickness, 99.995% purity) were purchased from Sigma-Aldrich Co. Potassium hydrogen carbonate (KHCO_3_, 99.5–101%), potassium chloride (KCl, 99.5–100.5%) and ethanol (C_2_H_5_OH, 96%) were purchased from PanReac. Potassium hydroxide (KOH, 85%) and ethanol absolute (C_2_H_5_OH, 98.8%) were purchased from Labkem. Vulcan XC-72 carbon powder (CAS N° 1333-86-4, N° 215-609-9) was purchased from FullCellStore. Nylon membrane filter (90 mm, 0.22 µm, Cod.MNY022090N) was purchased from FILTER-LAB ^®^. Cationic ion exchange membrane Nafion^TM^ 115 (CAS31175-20-9) was also purchased from Sigma-Aldrich. Carbon fiber paper Toray^TM^ (TP-90) was purchased from QuinTech. All chemicals were purchased with the highest analytical grade available and were employed without any further purification. All the solutions were prepared using MilliQ ultra-pure deionized water (DIW, 18.2 MW·cm^−1^ of resistivity) to avoid the presence of any trace metal impurities from water which could affect the CO_2_RR performance.

### 2.2. Synthesis Procedures

#### 2.2.1. In NPs Synthesis

In NPs were synthesized by modifying a simple two-step hydrothermal method [37]. Briefly, 0.110 g of the metallic precursor (InCl_3_) and 1 g of the capping agent (PVP) were dissolved in pure water at 60 °C under vigorous stirring for 30 min. Then, 0.230 g of the reductant agent (NaBH_4_), previously dissolved in cold DI water, was added to the solution, exposing an abrupt color change from translucent to dark grey as a result of the reduction of the metal particles. The resultant solution was maintained under continuous stirring and alternated with 3 min of sonication for another 30 min. Once a homogeneous solution is obtained, 50 mL of ethanol (96%) were added for inducing the precipitation of the particles. This step was repeated two times. The as-obtained product is filtered with a nylon membrane filter and washed with an ethanol/potassium hydroxide (0.1 M) solution (50/50 vol. %) or DI water/ethanol solution (50/50 vol%), depending on the catalyst batch prepared. The selection of these two different washing solutions was tested as route to optimize the cleaning step. Finally, the obtained wet powder was vacuum dried at 50 °C for 8 h. The as-obtained NPs were denoted as In_H2O_ and In_KOH_, respectively.

#### 2.2.2. In_x_/C_y_ Synthesis

In_x_/C_y_ NPs were synthesized using a similar methodology. Once the reductant agent is completely dissolved into the solution containing the indium precursor and the PVP (approximately after 30 min of continuous stirring), a proper amount of Vulcan XC-72 carbon powder was added to the mixture. Nominal metal loadings of 20 wt. % and 50 wt. %, respect to the carbon support were achieved and denoted as In_20_/C_80_ and In_50_/C_50_, respectively. Once the final suspension was stirred for 30 min and then sonicated for another 30 min, 50 mL of ethanol (96%) were added. Finally, the so-synthesized nanoparticles were filtered and washed with the same mixture of ethanol/potassium hydroxide and dried as described in the previous section.

#### 2.2.3. Catalytic ink and Working Electrodes Preparation

For the preparation of the In-based electrodes, a catalytic ink was firstly prepared by dispersing the In NPs in a Nafion solution (perfluorinated resin solution 5 wt.%) serving as a binder factor, with a catalyst/nafion mass ratio of 70:30 and diluted to 2 wt. % in absolute ethanol (98.8%). The solution was sonicated for 60 min and sprayed over a carbon paper (TP-090, Quintech) by means of an air brushing technique. To promote the deposit of uniform layers of ink on the cathode, the carbon substrate was set between two iron plaques placed on a hot plate at 110 °C to allow fast evaporation of the solvent. The catalytic area of the electrodes was 9 cm^2^ with the same mass loading in all of them of 0.8 mg·${\mathrm{cm}}_{\mathrm{geo}}^{-2}$ of In.

### 2.3. Physicochemical Characterization of Materials

XRD was carried out to identify the existence of crystalline phases in the samples. Patterns were recorded on a Linxeye-XE-D8-Advance spectrometer with a Cu-Kα radiation (1.54060 Å) and a source operating at 40 kV and 40 mA. To evaluate the surface morphology of the NPs, SEM was performed on a Hitachi-S4800 microscope operating at 0.5–30 kV. The average size of nanoparticles and plane spacing was study in detail by means of TEM and HR-TEM. The measurements were performed by a JEOL 2100Plus microscope operated at 200 kV, coupled with a EDX X-Max 80 T (Oxford Instruments). To detect the surface states of the metallic nanoparticles, XPS measurements were carried out in a customized system incorporating a hemispherical analyzer (SPECS Phoibos 150) and a non-monochromatized X-ray source (Al Kα, 1486.6 eV; Mg Kα, 1253.6 eV), working At 14.0 Kv of anode voltage and 14.4 anode current (200 W). The analyzer was operated at a fixed transmission and 20 eV pass energy with an energy step of 0.1 eV.

### 2.4. Electrochemical Characterization

The electrochemical tests were carried out at ambient temperature in a custom-made, two compartment sealed H-cell, separated by a proton exchange membrane (Nafion 115). The catholyte compartment was filled with 140 mL of a 0.5 M KHCO_3_ and 0.45 M KCl solution, continuously bubbled with Ar or CO_2_, during the cyclic voltammetry measurements. A constant purge of CO_2_ was maintaining during the electrolysis experiments to keep CO_2_ saturation and solution convection. The current densities obtained were normalized by the catalytic geometric area of the working electrodes (3 cm^2^ for In-based cathodes). A commercial AgCl/Ag (3.5 M KCl, Metrohm, Herisau, Switzerland) was used as a reference electrode. The anolyte compartment was filled with a 1 M KOH solution and a nickel mesh was used as counter electrode. Cyclic voltammetry sweep (CVS) and chronoamperometric measurements were carried out by means of a potentiostat PGSTAT302N system (Metrohm Autolab B. V., Utrecht, The Netherlands).

### 2.5. Products Analysis

Liquid aliquots were collected from the catholyte compartment every 30 min. To measure the formate concentration, we used ion chromatography (Dionex Easion) from the company Thermo Scientific (Waltham, MA, USA) with a suppressed conductivity detection, ASRS self-regenerating suppressor (Dionex ASRS ™ 300) and AS23 (Dionex IonPac™ AS23) column. A 4.5 mM Na_2_CO_3_ and 0.8 mM NaHCO_3_ was used as eluent. Formate concentration was obtained using different calibration curves over the 0.2 to 24 ppm range. FE for formate was calculated as:

(1) $$\mathrm{FE}\left(\%\right)=\frac{z\cdot F\cdot n}{Q}\cdot 100\;,$$

where z is the number of electrons exchanged for the reduction of CO_2_ to formate, F is the Faraday constant (96.485 C·mol^−1^), n is the number of formate moles present in the sample, and Q is the total charge transfer.

## 3. Results and Discussion

### 3.1. Materials Characterization

#### 3.1.1. XRD Diffraction Patterns and XPS Spectra

**Figure 1:**
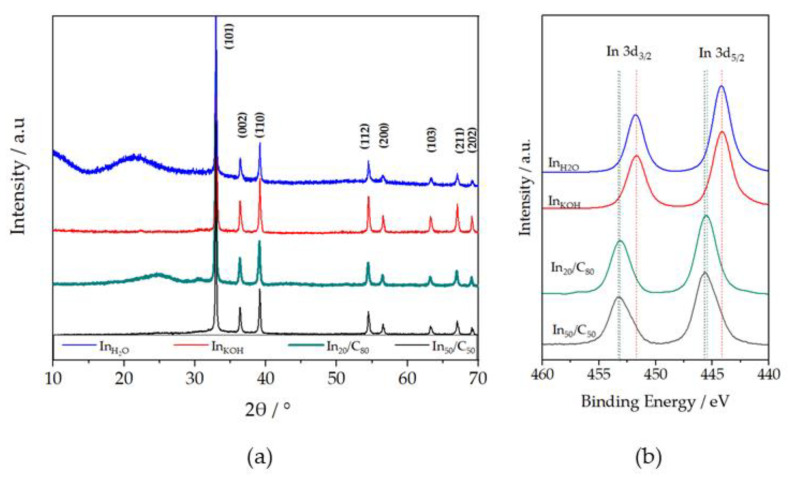
(**a**) X-ray diffraction patterns of In_H2O_, In_KOH_, In_20_/C_80_ and In_50_/C_50_ NPs, with lattice planes identification of mayor peaks [38,39]; (**b**) XPS spectra of In 3d for In_KOH_, In_20_/C_80_ and In_50_/C_50_ NPs.

The crystal structures of the as-synthesized samples were determined by XRD analyses. Figure 1a shows the diffraction patterns of the bulk In catalysts washed with H_2_O and KOH (In_H2O_ and In_KOH_, respectively), and In supported on carbon catalysts at different ratios (In_20_/C_80_ and In_50_/C_50_). All the samples analyzed exhibited sharp and well defined peaks at 32.96, 36.35, 39.14, 54.55, 56.53, 63.20, 67.01 and 69.07°, which, respectively, corresponded with the (101), (002), (110), (112), (200), (103), (211) and (202) crystallographic planes of a tetragonal crystalline phase of metallic In (COD:96-210-0457) [38,39,40]. As can be observed, the most intense peak was associated with the plane (101) of In, revealing that this plane was the most abundant in the In NPs in all cases [31]. Besides the sharp XRD peaks, a trace amounts of some impurities can be observed in the bulk In catalyst washed with water (In_H2O_), evidenced by the presence of a wide and low intensity peak at ca. 20°.

The broad peak at ca. 25° in the XRD pattern of In_20_/C_80_ can be ascribed to the (002) plane of C, in line with previous works [38,41,42]. On the other hand, the average crystal sizes of the catalysts were obtained from Scherrer’s equation [43]. The crystallite size obtained were 59.3 nm, 68.6 nm, 42.5 nm and 54.9 nm for InH_2_O, In_KOH_, In_20_/C_80_ and In_50_/C_50_ NPs, respectively.

To compare the composition of the catalysts surface of unsupported and supported, XPS analyses were carried out. The samples unsupported, In_H2O_ and In_KOH_, showed a doublet (Figure 1b), corresponding to In 3d_3/2_ and In 3d_5/2_ levels, with maxima for 3d_5/2_ signal at ca. 444.2 eV. On the other hand, In_20_/C_80_ and In_50_/C_50_ exhibited its 3d_5/2_ signal at ca. 445.5 and 445.6 eV, respectively. Although these values are slightly higher than those normally reported for and In^0^ (443.8 eV) and In^3+^ (445.0 eV) [44,45], this shift has been also found in some cases [12]. In fact, the calibration has been based on an interactive assignment of the binding energy for In3d peaks, considering reasonable values for that doublet, but a variation of around ±0.4 eV for In^0^ and ±0.6 eV for In^3+^ could be considered [46,47]. In addition, some binding energies displacement could be considered as consequence of the interactions with the support [48,49,50,51]. Therefore, the peaks at 445.5 for In_20_/C_80_ or 445.6 for In_50_/C_50_, and 444.5 eV can be ascribed to In^3+^ and In^0^ species, respectively. These results indicate the absence of oxidized species on the In_H2O_ and In_KOH_ materials, whereas carbon-supported samples contain a significant amount of oxidized In species. Furthermore, the slight displacement between the signals of the supported samples may suggest that In_50_/C_50_ sample contain a higher concentration of oxidized species than In_20_/C_80_, which is logical since the In loading is much higher in the former than in the latter. XPS measurements also revealed the presence of very weak nitrogen signals, which are likely to originate from PVP (Figure S1). The very low intensity of these signals indicate that N is present at trace levels on the surface of the catalysts, suggesting that the synthesis route used herein was effective in removing impurities. Thus, XRD and XPS data seem to point out the presence of In NPs with a surface shell enriched in In^3+^ species (as derived from XPS results) and a core mainly composed on metallic In (as revealed in XRD diffractograms) in carbon-supported materials.

#### 3.1.2. SEM

**Figure 2:**
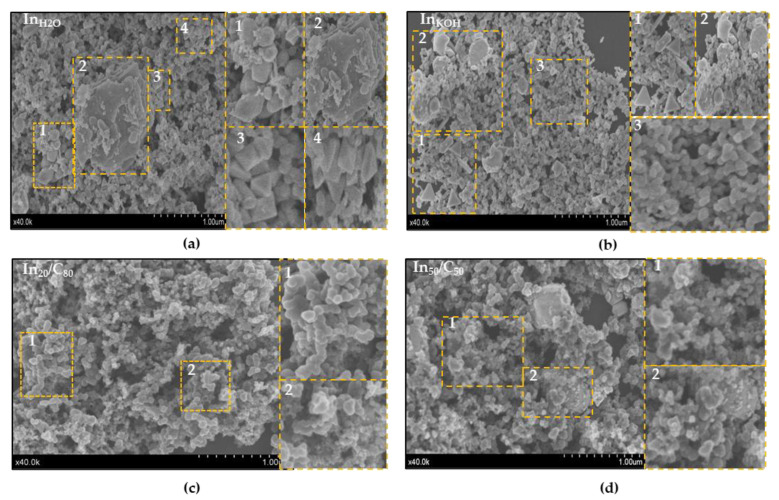
Representative SEM images of: (**a**) In nanoparticles washed with H_2_O; (**b**) In nanoparticles washed with KOH. In nanoparticles supported on carbon: (**c**) 20/80 wt. %; (**d**) 50/50 wt. %. Some enlargements of the SEM images are included for facilitating the interpretation.

The morphology of the samples was analyzed by means of SEM. A representative SEM image (Figure 2) shows the presence of diverse morphologies depending on the catalyst. As shown in Figure 2a, In_H2O_ exhibits a variety of shapes such as spheres, triangles, octahedrons, nanorods-like and also some bulk-like shapes. In_KOH_ in Figure 2b, for its part, also shows the presence of different morphologies, with a predominance of nearly spherical particles. The prevalence of spherical nanoparticles and the presence of triangles and also rod-like particles have been previously reported to be formed in reductive environments, pointing out that the reduction kinetics (represented by the addition rate of the reductant agent), as an influent factor in the shape of the nanocrystals [52]. Moreover, these mixed morphologies have been also reported as a function of synthesis time based on the existence of separate defined nucleation steps with different kinetics. It seems that, in early stages, spherical shapes dominate the particle population with a small presence of triangles but, as the synthesis time advances, the size of the spherical particles grows while the fraction of triangle increases [53]. Likewise, In NPs in the supported materials (In_50_/C_50_ and In_20_/C_80_) also exhibited a variety of shapes but with a higher fraction of spherical nanoparticles as compared to the unsupported samples (Figure 2c,d). It is known that the nature of the support could affect the dispersion of the supported metal and, consequently, its morphology and/or size [51]. In addition, the presence of the carbon support during the chemical reduction process might alter the rates of nucleation and growth of In nanoparticles, thereby favoring the formation of nanospheres versus other morphologies. One of the main factors determining the final morphology and size of metal nanoparticles prepared by the borohydride reduction method (the method used herein) is the rate of addition of the reductive agent. We believe that the presence of a porous material such as carbon might result in borohydride being adsorbed, thereby leading to more controlled and slower addition of the reductive agent to the reaction medium. The same reasoning is valid for the In metal precursor, which is likely to be adsorbed on the carbon material during the synthesis, reducing the available concentration in solution. We believe that these conditions (i.e., low metal precursor concentration and controlled and slow addition of the reductive agent) are particularly favorable for the isotropic growth of In nanoparticles, thereby leading to the formation of spherical shapes. It is worth mentioning that most of previous works reported the formation of unique geometries separately [52,54,55,56,57]. The synthesis method described in this work leads to a heterogeneous mixture of geometries, which can be an advantageous way to introduce edges sites and to enrich the catalyst morphology. This, considering the high structure sensitivity of the CO_2_RR, could represent a simple way for enhance the performance of the reaction.

#### 3.1.3. TEM

**Figure 3:**
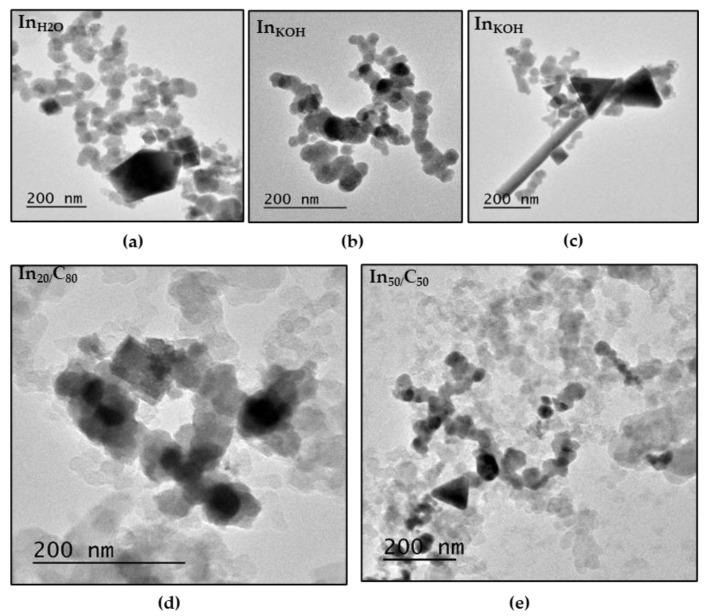
Structural analysis of the nanoparticles by TEM: (**a**) In NPs washed with H_2_O; (**b**,**c**) In NPs washed with KOH; (**d**) In over carbon with a ratio of 20/80 wt. %. (**e**) In over carbon with a ratio of 50/50 wt. %.

Likewise, the SEM analyses, TEM images also reveal the presence of mostly spherical particles (Figure 3a–e). The average size of the prevailing spherical nanoparticles is similar in all the samples (ca. 35 nm), although slight differences can be observed. The In_KOH_ catalyst showed spherical nanoparticles slightly larger (38.39 nm) than those of the In_H2O_ catalyst (35.95 nm). This difference could be attributed to the more effectiveness of the potassium hydroxide than water for removing the rest of the synthesis reagents. As was previously described in the XRD section, the sample washed with water exhibit the presence of some impurities remaining. Thus, it could be possible that the hydroxide ion may compete with the capping agent for be absorbed on the particle surface, which could induce an accelerated growth of the crystal by decreasing its surface energy [58]. It is worth mentioning that TEM images also confirmed the presence of difference morphologies. Thus, triangles, nanorods and octahedral particles of ca. 145 nm, 700 nm and 82 nm in size, respectively, can be clearly observed in the In_KOH_ catalyst (Figure 3c). However, as shown in Figure 3b, this sample also contained a significant number of spherical In nanoparticles, which are lower in size compared with the previous ones. While we do not rule out that In nanoparticles with special morphologies (e.g., triangles, nanorods and octahedral) have an effect on the overall catalytic activity, we believe that this effect is minor compared with that of spherical nanoparticles since they are lower in size and possess a higher fraction of high-energy edge and corner sites. These highly energetic sites have been reported to show higher activity than terrace atoms, which are prevalent in large nanoparticles with other morphologies such as nanorods and triangles [22]. For its part, the average sizes of the spherical nanoparticles of In_20/_C_80_ and In_50/_C_50_ were 30.67 nm and 36.47 nm, respectively. Future studies are necessary to evaluate the possibility of enhancing the electroactivity of the samples by controlling the morphology and size of the In particles. A possible approach could be modifying the addition rate of sodium borohydride and the reaction time, both parameters being reported as main factors involved in the nucleation kinetics under reductive environments [52,53]. Additionally, the smaller size of the In_20/_C_80_ NPs confirms the role of the support on the nucleation mechanism of the indium nanoparticles suggested in the previous section.

**Figure 4:**
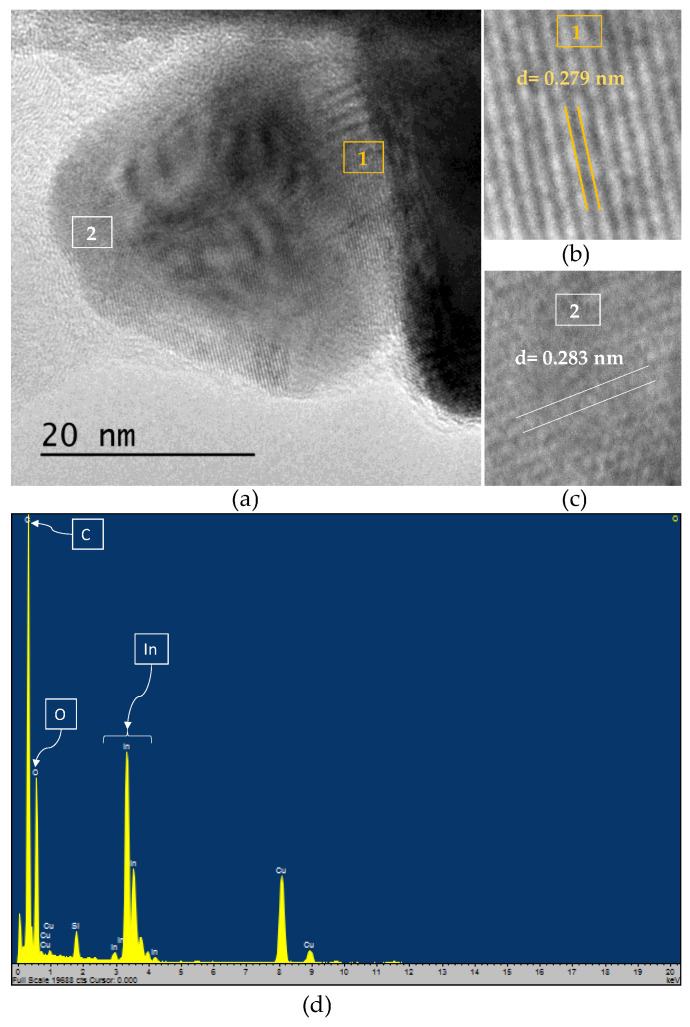
(**a**) HR-TEM micrograph of In_50_/C_50_ nanoparticles; (**b**,**c**) detail of lattice fringe spacings on spherical nanoparticles; (**d**) EDX pattern showing the elemental composition of the nanoparticles.

To confirm the presence of oxygenated species (In^3+^), we analyzed the spherical particles of In_50_/C_50_ by HR-TEM (Figure 4). As shown in Figure 4a, several lattice fringe spacings can be observed around and inside the spheric nanoparticle. In the HR-TEM image, the lattice fringe of 0.279 nm (Figure 4b) can be assigned to the (101) plane of metallic In which is in agreement with that observed by XRD analyses [35]. In addition, the lattice fringes with interplanar spacing of ca. 0.281 nm (Figure 4c) can be indexed to the (220) planes of In(OH)_3_ [47,58]. These results confirm the existence of oxygenated species (In(OH)_3_) on the catalyst surface, in accordance with that suggested by XPS. Moreover, the element distributions were determined by energy-disperse X-ray spectroscopy (EDX) analyses (Figure 4d). As expected, two intense C and In signals for can be observed since the catalyst is mainly formed by these two elements. In addition, a noticeable peak of O is also evident, revealing the presence of an oxidized phase which may be related with the formation of a thin surface layer [22]. Despite the existence of oxidized species has been probed by XPS and HR-TEM, the signals of In(OH)_3_ in the XRD cannot be observed, which can be ascribed to the existence of this specie as an essentially amorphous phase. Hence, according to XRD, XPS and HR-TEM data, the In over carbon particles are mainly composed by metallic indium along with an oxidized layer at the surface, possibly of In(OH)_3_.

### 3.2. Electrochemical Characterization

#### 3.2.1. Effect of the Washing Agents

**Figure 5:**
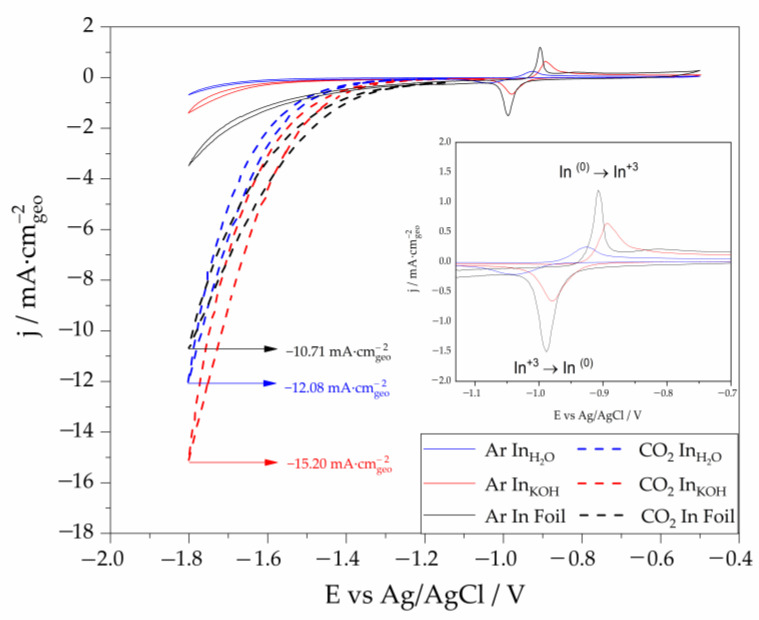
Cyclic voltammograms obtained in Ar (solid lines) and CO_2_ (dashed lines) saturated solutions (KHCO_3_ 0.5 M + KCl 0.45 M) for In_H2O_, In_KOH_ and In foil electrodes. Scan rate 50 mV·s^−1^.

The electrochemical characterization of the In_H2O_ and In_KOH_ NPs supported on carbon Toray paper were carried out by means of CVS. The materials were screened between −0.5 V and −1.8 V vs. Ag/AgCl reference electrode, in a H-cell saturated with Ar/CO_2_ with a KHCO_3_ 0.5 M + KCl 0.45 M solution. Figure 5 shows representative CVS experiments for each material and for an indium foil for sake of comparison, under Ar and CO_2_ saturated conditions. Ar-saturated CSV (solid lines) shows the redox pair corresponding with the indium species formed on the interface and the current densities associated with the oxidation/reduction processes. As can be observed (see inset graph in Figure 5), there is an anodic peak which corresponds to the characteristic oxidation of In^0^ to In^3+^ at −0.92 V for In_H2O_, −0.89 V for In_KOH_ and −0.91 V for In foil. The presence of this peak suggests the formation of a passive film of In_2_O_3_ or In(OH)_3_ [38,59]. On the other hand, the reduction of In^3+^ to In^0^ can be observed in the negative sweep going at −1.04 V, −0.98 V and −0.99 V, for In_H2O,_ In_KOH_ and In foil, respectively. Additionally, the current densities of the anodic and cathodic peaks for In_KOH_ sample are higher than those obtained for In_H2O_ NPs, revealing a lower reducibility of In particles in In_H2O_ as compared to the In_KOH_ catalyst [22]. Furthermore, it is well known that, as the potential shifts to more negative values, the hydrogen evolution reaction (HER), which competes with CO2RR, becomes more pre-dominant. As shown in Figure 5, under Ar conditions, the In foil electrode showed higher current densities than the In_KOH_ and In_H2O_ samples at low voltages (−1.4 V and lower), indicating a higher activity of the foil sample towards the HER. These results are in line with previous works revealing In foil to be particularly active in promoting the HER versus CO2RR [22,35,59]. In terms of onset potentials, the In foil sample showed more positive values (−1.4 V) than the In_KOH_ (−1.7 V) and In_H2O_ (−1.8 V) samples, which is also in agreement with the foil sample having a higher HER activity.

On the other hand, the CO_2_RR performance of In_KOH_, In_H2O_ and the In foil has been evaluated in a solution saturated with CO_2_ on the potential range from −1.15 V to −1.8 V (represented with dashes in Figure 5). The change in the pH once CO_2_ saturates the solution (pH_CO2_ = 7.42) in contrast to that when using Ar (pH_Ar_ = 9.33), results in a shift to more negative potentials at which the reduction processes take place. Thus, the reduction onset potentials for In_KOH_, In_H2O_ and In foil were −1.35 V, −1.42 V and −1.25 V vs. Ag/AgCl, respectively [22,38]. In_KOH_ NPs reached the highest current density at −1.8 V vs. Ag/AgCl (−15.20 mA·cm_geo_^−2^) as compared to that obtained by In_H20_ (−12.08 mA·cm_geo_^−2^) and the In foil (−10.71 mA·cm_geo_^−2^). In nanoparticles washed with KOH exhibit a better performance than pure In foil, despite the former has a much lower metallic loading, due principally to the active surface area of the nanoparticles is greater than the bulk material. This promotes contact between the catalyst and the reactants, leading to an enhanced CO_2_ reduction. In addition, In_KOH_ NPs also exhibit a larger current density and a lower onset potential than the In_H2O_ NPs. Despite the clear environmental advantages derived from the use of water, it seems that it is necessary to use a more “aggressive” washing agent than just pure H_2_O to clean completely the catalyst surface. As previously commented, XRD results showed that some impurities remain in the In NPs when water was used during the washing step and that could hinder the electroactivity of these nanoparticles. At the same time, some big conglomerates are observed in SEM images, which will also affect negatively the electroactivity of the material.

#### 3.2.2. Effect of the Carbon Support

**Figure 6:**
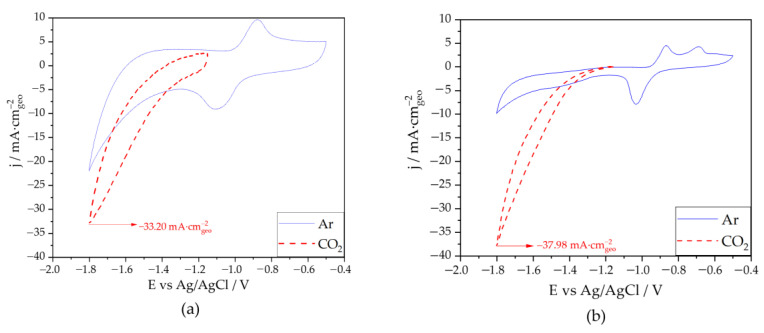
Cyclic voltammograms obtained in Ar (solid lines) and CO_2_ (dashed lines) saturated solutions (KHCO_3_ 0.5 M + KCl 0.45 M) for: (**a**) In_20_/C_80_ and (**b**) In_50_/C_50_ NPs. Scan rate 50 mV·s^−1^.

Based on the results obtained from the comparison of the effectiveness of the washing agents, In nanoparticles washed with KOH were supported on carbon Vulcan following the synthesis method described previously. To study the effect of supporting In on carbon, two different catalysts were electrochemically characterized, In_20_/C_80_ (Figure 6a and Figure S3a) and In_50_/C_50_ (Figure 6b and Figure S3b). As expected, the experiments carried out with Ar (solid line) showed the characteristic patterns for the oxidation (positive going sweep) and for the reduction (negative going sweep) of indium species. In_20_/C_80_ catalyst response under Ar saturation conditions shows an anodic peak at −0.88 V and a cathodic peak at −1.10 V. However, In_50_/C_50_ electrode displayed two anodic peaks at −0.69 and −0.87 V, unlike In_KOH_ and In_20_/C_80_. The peak at −0.87 V corresponds to the oxidation of In^0^ to In^+3^ species, while the peak at −0.69 V may be associate to the presence of In^+^ as a product of an intermediate kinetic step. This may suggest that In_50_/C_50_ NPs exhibit a lower kinetic response than those of In_KOH_ and In_20_/C_80_ NPs at the same potential window [60,61,62]. On the other hand, for CO_2_ saturated solutions, the electrochemical response of the catalysts supported on carbon, in terms of current density, is more than double of those obtained for unsupported catalysts at −1.8 V vs. Ag/AgCl (see Table 1). This behavior can be attributed to a higher double layer capacitance (cdl) of the carbon-supported samples (Figure S2 and Table S1). Thus, the carbon-supported samples showed higher cdl values (26.5 and 6.4 mF·cm^−2^ for In20/80 and In50/50, respectively) than the unsupported In_KOH_ (0.05 mF·cm^−2^). The presence of porous carbon, thanks to its high ratio surface/volume, can increase the capacitance of the double layer, resulting in higher charge-storage capacities at the interface and improved CO_2_RR performance [63]. However, the large increase in activity of the In50/C50 catalyst could be be ascribed to the presence of an oxidized layer on the catalyst. The higher concentration of In(OH)_3_ at the surface in the In_50_/C_50_ (see XPS and HR-TEM results) would explain the better CO_2_RR performance of this catalyst over the rest of them (see summary Table 1.) [22]. Oxidized metal species have been previously reported to promote both the activity and selectivity in CO_2_RR by facilitating proton transfer steps [35,64]. With regard to indium catalysts, In^0^, In_2_O_3_ and In(OH)_3_ have been proposed as the actual active sites for CO_2_RR [21,22]. The improved electroactivity of these materials was ascribed to the formation of carbonate species by the interaction of CO_2_ with the surface oxidized layer [65]. Surface In^+3^ species (e.g., In(OH)_3_) have been reported to allow the chemisorption of CO_2_ via formation of In-CO_3_^−^ species. These species are reduced at negative potentials (via a two-proton and two-electron process) to form HC(OH)_2_O^−^ intermediates, which are further released as HCOOH from the electrode interface [22,59]. Furthermore, the influence of a higher concentration of In(OH)_3_ in In_50_/C_50_ seems to predominate over the decrease observed in the particle size for the In_20_/C_80_ in terms of current densities. In addition, it can be also observed in Figure 6a,b that the onset potential at which the CO_2_ reduction takes place for the carbon supported catalysts shift to less negative values, which is a clear advantage concerning electric consumption saving issues.

#### 3.2.3. Effect of the Applied Potential

**Figure 7:**
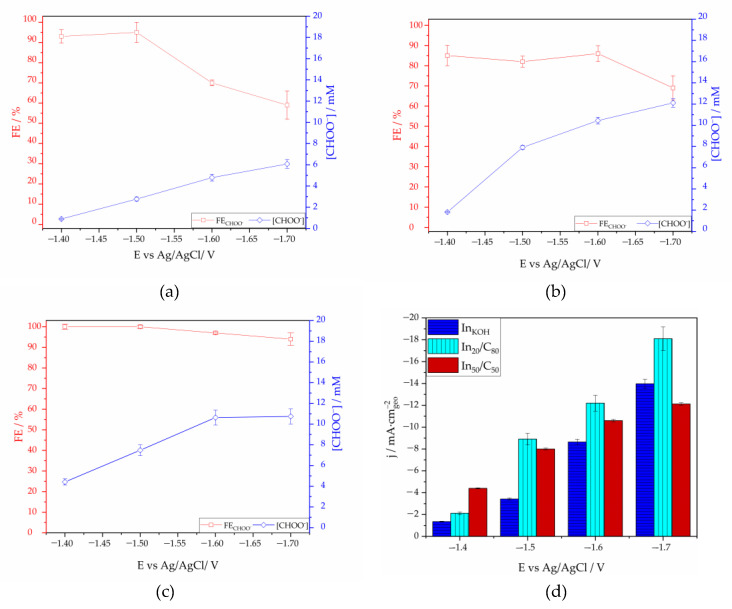
Faradaic efficiency to formate vs. cathodic potential and mM of formate produced for: (**a**) In_KOH_; (**b**) In_20_/C_80_; (**c**) In_50_/C_50_ catalyst; and (**d**) average current densities as a function of the potential.

The CO_2_RR performance of In_KOH_, In_20_/C_80_ and In_50_/C_50_ was screened in a potential range between −1.4 V and −1.7 V vs. Ag/AgCl in a H-cell under continuous CO_2_ bubbling in a solution of 0.5 M KHCO_3_ and 0.45 M KCl. As shown in Figure 7a–c, formate was the main product in all cases. There is a voltage window (from −1.40 to −1.50 V) over which formate can be generated with very high FE values in all cases. Particularly, In_50_/C_50_ exhibited the highest Faradaic efficiency towards formate at any cell potential, with values near 100% at −1.4 V and −1.5 V, and with a slight decrease (97%) at −1.6 V vs. Ag/AgCl. The FE to formate decreased as the voltage became more negative, particularly for the In_KOH_ and In_20_/C_80_ samples, probably due to the generation of hydrogen via HER at the high negative voltages. The In_50_/C_50_ sample also showed higher current density values than the rest of samples over a wide range of potentials.

As mentioned previously, the higher FE to formate observed for In_50_/C_50_ can be attributed, among other aspects, to the presence of surface In(OH)_3_ species leading to the formation of essential intermediates for formate production. In particular, at −1.6 V, In_50_/C_50_ catalyst demonstrated approximately 28% of FE increase in comparison with In_KOH_, and almost 10.5% with In_20_/C_80_. Analyzing the total current densities as a function of the potential (Figure 7d) it can be observed that In_KOH_ exhibit an obviously lower FE to formate in comparison to the In supported on carbon samples. This can be ascribed to the absence of oxidized species in In_KOH_, as suggested by XPS results.

The total current density is also higher for In_20_/C_80_ than for In_50_/C_50_ at a potential range between −1.5 V and −1.7 V vs. Ag/AgCl. However, considering FE to formate (lower of 90% at any potential), is probable that the In_20_/C_80_ promotes the HER reaction at any potential due to the low concentration of oxidized species, increasing the total current densities registered. It seems that the higher concentration of carbon facilitates the HER, increasing the total current density, but resulting in a detrimental effect on the formate production. These results are in accordance with that suggested in previous works [22,66].

The optimum cataIyst In50/C50 showed nearly complete selectivity to formate while maintaining an acceptable current density of 10.6 mA. cm^−2^. Compared with other In-based catalysts reported in the literature (Table 2), In50/C50 showed slightly higher FE to formate and comparable current density values.

#### 3.2.4. Electrodes Stability

**Figure 8:**
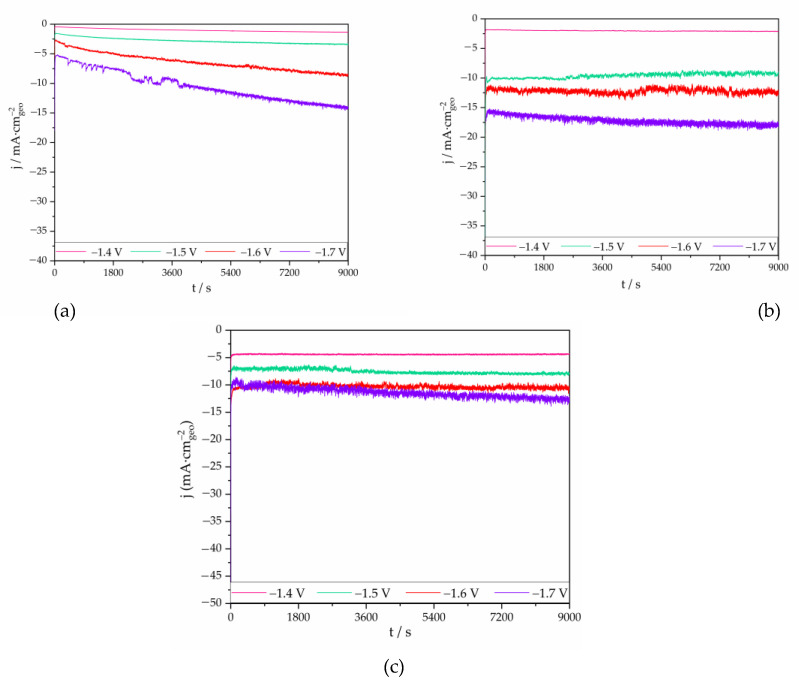
Chronoamperometric measurements at −1.4, −1.5, −1.6 and −1.7 V vs. Ag/AgCl, for: (**a**) In_KOH_; (**b**) In_20_/C_80_; (**c**) In_50_/C_50_ catalyst.

The durability (stability of the current density as a function of time) of the In nanoparticles (In_KOH_, In_20_/C_80_ and In_50_/C_50_) was studied performing long-term electrolysis at different potentials (Figure 8). It is worth mentioning that at −1.4 V, where the current density is almost negligible for In_KOH_ and In_20_/C_80_, In_50_/C_50_ catalyst shows approximately 5 mA·cm^−2^. That could be considered as a preliminary indication of the higher activity of this catalyst towards CO_2_RR, even at a such low potential as compared to the rest of catalysts analyzed. The In_KOH_ catalyst showed an evident decay on the current density over time at −1.7 V, and even at −1.6 V. This can be explained because of the lower overpotential of the HER for this catalyst. In fact, this is in agreement with the data of FE shown previously, where it could be observed that FE to formate was below 70% (i.e., the hydrogen evolution is taking place markedly). However, In_20_/C_80_ and In_50_/C_50_ exhibit mainly stable current densities over time except for −1.7 V where, as mentioned, the hydrogen evolution reaction is favored. Hence, a superior behavior can be observed for the supported In NPs. As an example, the current stability for In_20_/C_80_ and In_50_/C_50_ catalyst remained essentially steady at −1.6 V vs. Ag/AgCl at about −12.5 mA·cm_geo_^−2^ and −10.6 mA_geo_·cm^−2^, respectively, for more than 2.5 h. Evidently, these results proof that the addition of carbon to the In nanoparticles not only increases the activity and selectivity of the CO_2_RR, but also has a positive effect into the stability of the catalyst, which is of vital importance for the scalability of the materials to more realistic applications.

**Figure 9:**
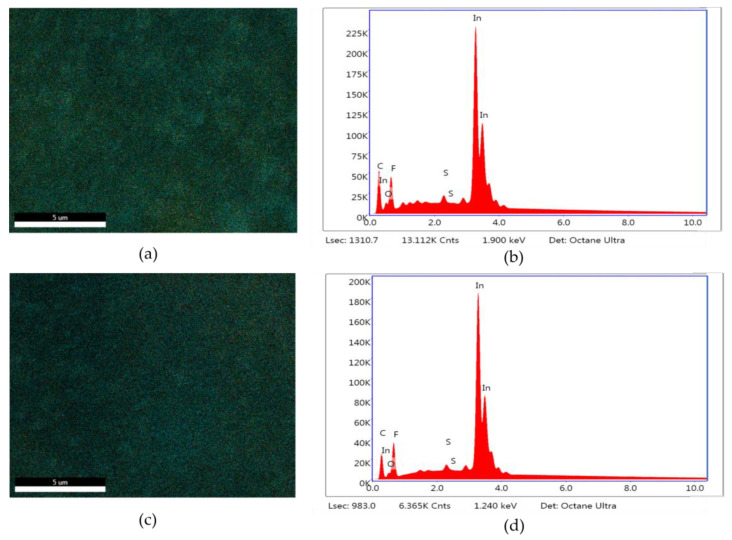
Pre-electrolysis: (**a**) In distribution mapping of the fresh In_50_/C_50_ electrode surface; (**b**) EDS measurements of the fresh In_50_/C_50_ electrode; post-electrolysis: (**c**) In distribution mapping of In_50_/C_50_ electrode surface after CO_2_RR; (**d**) EDS measurements of the In_50_/C_50_ electrode after CO_2_RR.

The stability of the optimum In_50_/C_50_ catalysts was further evaluated by performing SEM elemental mapping for In and EDS measurements before and after 2.5 h of reaction. As shown in Figure 9a,c, In remained uniformly distributed on the electrode surface after reaction, with no evidence of agglomerations of significant losses. Moreover, the EDS measurements before and after reaction (Figure 9b,d) were very similar, indicating that the electrode remained stable after reaction.

**Figure 10:**
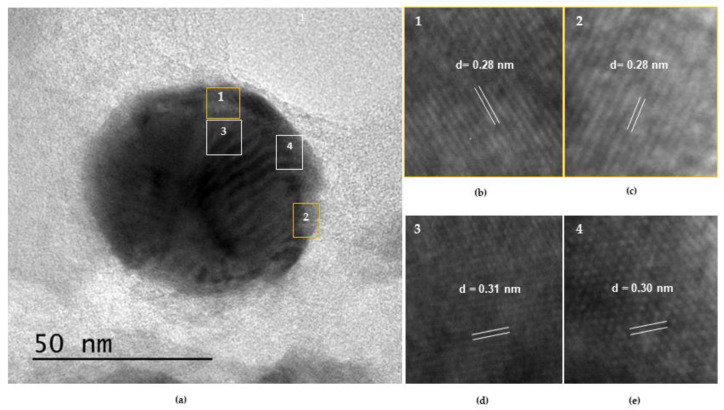
(**a**) HR-TEM micrograph of particle deposited on In_50_/C_50_ electrode, measure after 2.5 h of electrolysis. (**b**–**e**) detail of lattice fringe spacings on spherical nanoparticle.

On the other hand, HR-TEM analyses were carried out to corroborate the presence of planes ascribed with In^+3^ species by measuring the lattice fringe spacings in the particles deposited on the electrode after 2.5 h of reaction. As shown in Figure 10a, several lattice fringe spacings inside and around the deposited In particle can be observed. These lattice spacings at different points of the particle revealed values of ca. 0.28 nm and 0.31 nm (Figure 10b–e), characteristics of the planes (220) and (121) planes of In(OH)_3_, respectively. These results are line with the results exposed in Section 3.1.3. Additionally, the lattice fringe around 0.31 nm could be indexed to (121) plane of In(OH)_3_. These results suggest that oxidized In species remained stable as active sites during the reaction.

**Figure 11:**
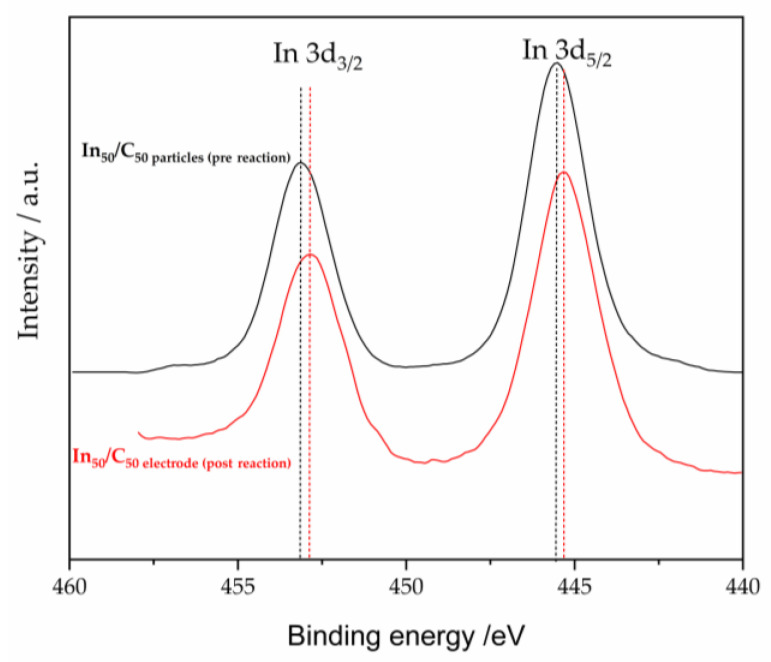
XPS spectra of In 3d for In_50_/C_50_ NPs pre reaction and electrode post reaction.

Additionally, the surface estate of the electrode was studied by XPS. Figure 11 compares the XPS spectra of the In_50_/C_50_ electrode after reaction with that of In_50_/C_50_ particles before reaction. As shown in Figure 11, the XPS spectra of the electrode after reaction was nearly similar to that of the fresh material. A slight shift between both spectra was observed, which can be ascribed to the interactions on In nanoparticles with the support. Consequently, based on the XPS data, we can infer that the In_50_/C_50_ electrode mostly maintained its initial properties after 2.5 h of electrochemical reaction under highly reductive potentials.

## 4. Conclusions

In this study, a simple and scalable method to obtain heterogeneous indium nanoparticles and carbon-supported indium nanoparticles under mild conditions is described in detail. These samples were tested in the electroreduction of CO_2_ reaction. Physicochemical characterization revealed all the samples to be composed of In nanoparticles with heterogeneous morphologies, mostly spherical. As revealed by XPS, the carbon-supported samples contained oxidized In species along with reduced In, while the unsupported samples only contained metallic In^0^ species. Compared to the unsupported samples, the carbon-supported materials showed higher electrochemical activities and selectivities to formate. The optimum catalyst obtained by this synthesis method (i.e., In_50_/C_50_) showed a high faradaic efficiency to formate (ca. 97%) at −1.6 V vs. Ag/AgCl while showing a stable current density above 10 mA cm^−2^ for more than 2.5 h. The presence of oxidized species in the carbon-supported samples might be responsible for the improved performance of these samples. Additionally, it is recommended to perform more studies to evaluate the effect of different variables on control in morphology and size of NPs, and study in detail the role of carbon support over the catalyst properties.

## Figures and Tables

**Table 1:** Current densities (mA·cm^−2^) of the different catalysts studied obtained in a CO_2_ saturated solution.

Catalyst	Particle Size (nm)	Current Density at −1.8 V vs. Ag/AgCl (mA·cm_geo_^−2^)	Onset Potential of CO_2_RR (V) vs. Ag/AgCl
In foil	-	−10.71	−1.25
In_H2O_	35.95	−12.08	−1.35
In_KOH_	38.39	−15.20	−1.42
In_20_/C_80_	30.67	−33.20	−1.20
In_50_/C_50_	36.47	−37.98	−1.20

**Table 2:** Performance of In-based catalyst in the CO_2_RR.

Electrocatalyst	Electrolyte	E (V)	j (mA. cm^−2^)	FE _HCOOH_	Ref.
In_50_/C_50_	0.5 M KHCO_3_ +0.45 M KCl	−1.6 V vs. Ag/AgCl (−0.96 V vs. RHE)	−10.6	~97%	This work
In/C (mp-in)	0.1 M KHCO_3_	−0.95 V vs. RHE	−29.6	~90%	[24]
In_2_O_3_@C	0.5 M KHCO_3_	−0.9 V vs. RHE	−29.5	~88%	[66]
In(OH)_3_/C	0.5 M K2_S_O_4_	−1.1 V vs. RHE	−5,2	~77%	[27]
In/C	0.1 M KHCO_3_	−1.0 V vs. RHE	−1	87.8%	[7]
In	0.1 M KHCO_3_	−1.55 V vs. RHE	5.0	94.9%	[67]
In NPs	0.5 M K_2_SO_4_	−1.5 V vs. Ag/AgCl	~6.0	~90%	[22]

## Data Availability

The data presented in this work is contained within this article or supplementary material. More detail can be requested from the authors.

## Supplementary Materials

The following supporting information can be downloaded at: https://www.mdpi.com/article/10.3390/nano13081304/s1, Figure S1: XPS survey data for the In_50_/C_50_ sample showing the signals for the C 1s, N 1s, O 1s and In 3d levels; Figure S2: CVS curves at scan rates from 10 to 90 mV·s^−1^ and capacitive current density against the scan rates and capacitive double layer (C_dl_) estimation of of In_KOH,_ In_20_C_80_ and In_50_C_50_; Figure S3: Cyclic voltammograms normalized by electrochemically active surface area (ECSA) obtained in Ar and CO_2_ saturated solutions (KHCO_3_ 0.5 M + KCl 0.45 M) for In_20_/C_80_ and In_50_/C_50_ NPs; Table S1: Double layer capacitance and ECSA calculation of the best materials synthesized: In_20_C_80_ and In_50_C_50_ [68,69,70].
