# Supplementary material for: Facile Synthesis of Heterogeneous Indium Nanoparticles for Formate Production via CO2 Electroreduction
Source: Nanomaterials (Basel). 2023 Apr 7;13(8):1304. doi: 10.3390/nano13081304 (PMC10142922; doi:10.3390/nano13081304)
Supplement: Supplementary file 1 [file nanomaterials-13-01304-s001.zip › nanomaterials-2219690-supplementary.pdf]

Supporting information: Facile synthesis of heterogeneous indium nanoparticles for formate production via CO_2 electroreduction

Ana Cristina Pérez-Sequera [1], Manuel Antonio Diaz-Perez [1], Mayra Anabel Lara Angulo [1], Juan P. Holgado [2] and Juan Carlos Serrano-Ruiz [1,*]

[1] Materials and Sustainability Group, Department of Engineering, Universidad Loyola Andalucía, Avda. de las Universidades s/n, 41704 Dos Hermanas, Seville, Spain; acperez@uloyola.es, malangulo@uloyola.es, madiaz@uloyola.es

[2] Instituto de Ciencia de Materiales de Sevilla and Departamento de Química Inorgánica, (CSIC-Univ de Sevilla), Av. Américo Vespucio, 49, 41092 Sevilla, Spain. jp.holgado@csic.es

* Correspondence: jcserrano@uloyola.com

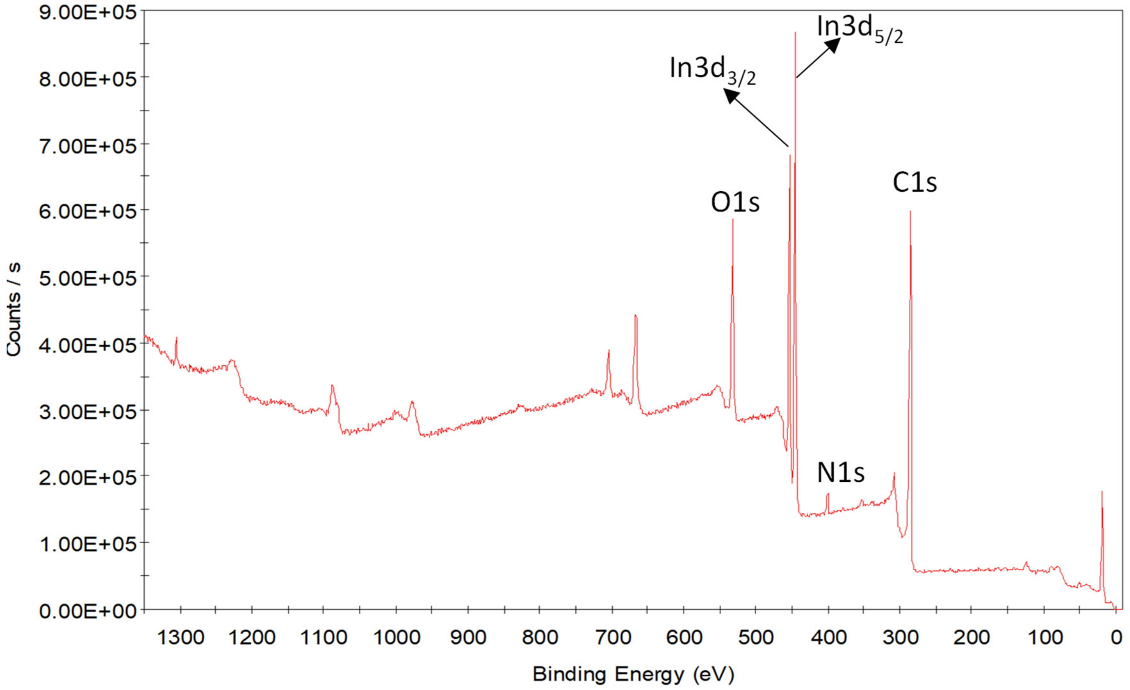

Figure S1. XPS survey data for the In_{50}/C_{50} sample showing the signals for the C 1s, N 1s, O 1s and In 3d levels.

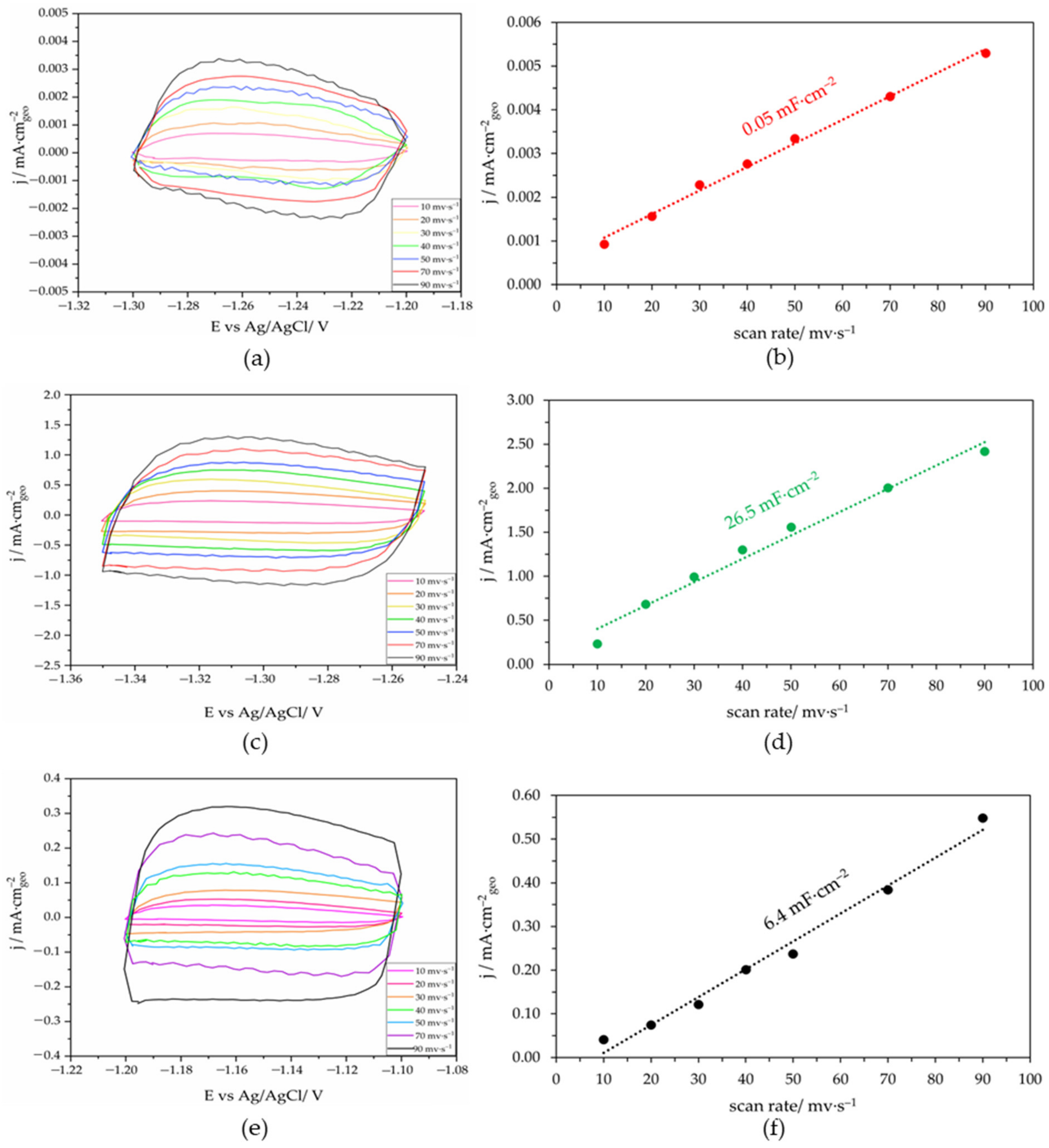

Figure S2. (a) CVS curves of In_{KOH} at scan rates from 10 to 90 mV·s^{-1}. (b) Capacitive current density against the scan rates and capacitive double layer (C_{dl}) estimation of In_{KOH}; (c) CVS curves of In_{20}/C_{80} at scan rates from 10 to 90 mV·s^{-1}; (d) Capacitive current density against the scan rates and capacitive double layer (C_{dl}) estimation of In_{20}/C_{80} ; (e) CVS curves of In_{50}/C_{50} at scan rates from 10 to 90 mV·s^{-1}; (e) Capacitive current density against the scan rates and capacitive double layer (C_{dl}) estimation of In_{50}/C_{50}.

The potential windows chosen for double layer capacitance measurements, correspond with ranges where no faradaic processes are observed. The capacitive current densities at -1.25 V for In_{KOH}, -1.30 V for In_{20}/C_{80} and -1,15 V for In_{50}/C_{50}, was plotted against the scan rate, and the resulting linear fitting was used to determine the specific capacitances of the materials.

The specific ECSA of the materials is calculated from the double layer capacitance (C_{dl}) following the next equation:

$$ECSA = \frac{S \cdot C_{dl}}{C_s}$$

Where S is equivalent to the geometric area of the working electrode and C_s is the specific capacitance of the sample or the capacitance of an atomically smooth planar surface of the material per unit area under identical electrolyte conditions. The value of C_s was 0.029 mF cm^{-2} based on the generally used values on literature [1–3]

Table S1. Double layer capacitance and ECSA calculation of the best materials synthesized: $In_{20}C_{80}$ and $In_{50}C_{50}$.

**Electrode**	**Double layer capacitance (C_{dl}) (mF·cm^{-2})**	**ECSA (cm^2)**
In_{KOH}	0.05	5.17
$In_{20}C_{80}$ NPs	26.5	2741.38
$In_{50}C_{50}$ NPs	6.4	662.07

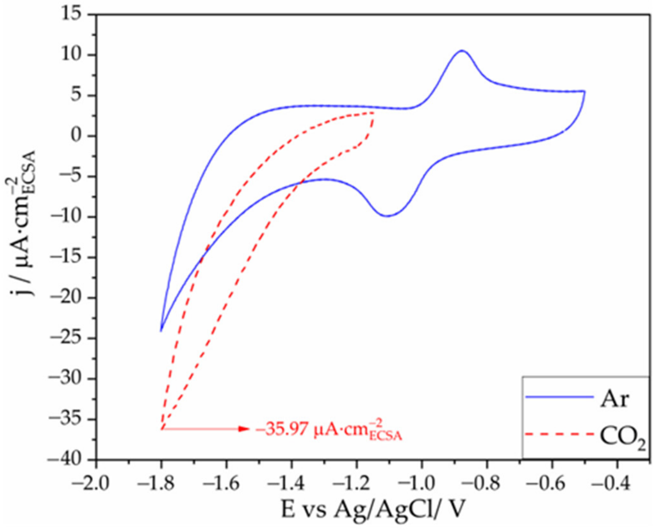

(a)

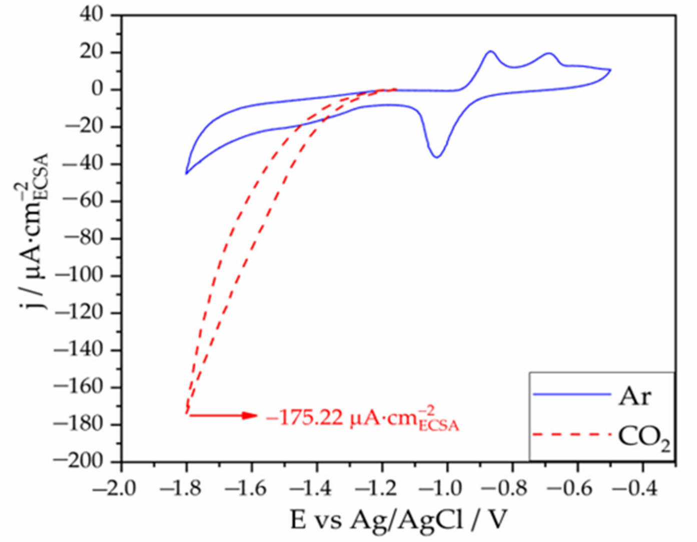

(b)

Figure S3. Cyclic voltammograms normalized by electrochemically active surface area (ECSA) obtained in Ar (solid lines) and CO_2 (dashed lines) saturated solutions ($KHCO_3$ 0.5 M + KCl 0.45 M) for: (a) In_{20}/C_{80} and (b) In_{50}/C_{50} NPs. Scan rate 50 mV·s^{-1}

References

1. Wang, J.; Ning, S.; Luo, M.; Xiang, D.; Chen, W.; Kang, X.; Jiang, Z.; Chen, S. In-Sn alloy core-shell nanoparticles: In-doped SnOx shell enables high stability and activity towards selective formate production from electrochemical reduction of CO2. *Appl. Catal. B Environ.* **2021**, *288*, 119979, doi:https://doi.org/10.1016/j.apcatb.2021.119979.

2. Li, C.W.; Ciston, J.; Kanan, M.W. Electroreduction of carbon monoxide to liquid fuel on oxide-derived nanocrystalline copper. *Nature* **2014**, *508*, 504–507, doi:10.1038/nature13249.

3. Zheng, X.; De Luna, P.; García de Arquer, F.P.; Zhang, B.; Becknell, N.; Ross, M.B.; Li, Y.; Banis, M.N.; Li, Y.; Liu, M.; et al. Sulfur-Modulated Tin Sites Enable Highly Selective Electrochemical Reduction of CO2 to Formate. *Joule* **2017**, *1*, 794–805, doi:https://doi.org/10.1016/j.joule.2017.09.014.
